# Evaluation of Antiproliferative, Antimicrobial, Antioxidant, Antidiabetic and Phytochemical Analysis of *Anogeissus dhofarica* A. J. Scott

**DOI:** 10.3390/antibiotics12020354

**Published:** 2023-02-08

**Authors:** Rabia Maqsood, Faizullah Khan, Saeed Ullah, Ajmal Khan, Habib Al-Jahdhami, Javid Hussain, Afaf M. Weli, Danial Maqsood, Shaikh Mizanoor Rahman, Amjad Hussain, Najeeb Ur Rehman, Ahmed Al-Harrasi

**Affiliations:** 1 Natural & Medical Sciences Research Center, University of Nizwa, Nizwa 616, Oman; 2 Department of Chemistry, University of Okara, Okara 56300, Pakistan; 3 Department of Pharmacy, Abdul Wali Khan University, Mardan 23200, Pakistan; 4 Department of Biological Sciences & Chemistry, College of Arts and Sciences, University of Nizwa, Nizwa 616, Oman; 5 School of Pharmacy, College of Pharmacy and Nursing, University of Nizwa, Nizwa 616, Oman; 6 Department of Chemistry, University of Lahore, Lahore 54590, Pakistan

**Keywords:** *Anogeissus dhofarica* A. J. Scott, cytotoxicity, antimicrobial, HR-ESI-MS, antidiabetic, phytochemical analysis, antioxidant

## Abstract

In the current study, methanol (ADAM) extracts and their fractions, including chloroform (ADAC), ethyl acetate (ADAE), n-hexane (ADAH), and aqueous (ADAA) fractions, were prepared from aerial parts of *Anogeissus dhofarica* and evaluated for phytochemical assessment, high-resolution electrospray ionization mass spectrometry (HR-ESI-MS) analysis, and in vitro bioassays. The qualitative analysis determined that, except alkaloids, all the representative groups were found to be present in the analyzed samples. Samples under quantitative study displayed the highest amount of total phenolic contents in the ADAE fraction, while total flavonoid contents were highest in the ADAM extract. The ADAM extract was subjected to HR-ESI-MS to identify the chemical constituents that presented twenty-two bioactive ingredients, outlined for the first time from *A. dhofarica,* mainly contributed by sub-class flavanones. In the case of antimicrobial activity, the ADAE extract revealed an effective zone of inhibition (ZOI) against the Gram-positive bacterial strain (*Staphylococcus aureus*) with an MIC value of 0.78 ± 0.3 mg/mL, while the ADAA extract exhibited higher ZOI (34 ± 0.12 mm) against the fungal strain *Candida kruzei* with an MIC of 0.78 mg/mL. In the DPPH (2,2-diphenyl-1-picrylhydrazyl) analysis, the ADAE extract exhibited a maximum scavenging potential with an IC_50_ of 9.8 ± 1.2 μg/mL, succeeded by the ADAM extract with an IC50 of 17.4 ± 0.4 μg/mL free radical scavenging capability. In the antidiabetic assessment, the ADAE extract was the most effective, with an IC_50_ of 6.40 ± 0.1 μg/mL, while the same extract demonstrated prominent activity with 30.8% viability and an IC_50_ of 6.2 ± 0.3 μg/mL against breast cancer cell lines. The brine shrimp lethality assay demonstrated a correlation with the in vitro cytotoxicity assay, showing the ADAE extract as the most active, with a 70% mortality rate and an LC_50_ of 300.1 μg/mL. In conclusion, all the tested samples, especially the ADAE and ADAM extracts, have significant capabilities for the investigated activities that could be due to the presence of the bioactive compounds.

## 1. Introduction

Since ancient times, the healing potential of plants has been thoroughly investigated. The therapeutic benefits of medicinal plants include their antioxidant, antibacterial, anticancer, antimalarial, analgesic, and antidiabetic properties [1,2]. Medicinal plants are mostly used because they are easily available and relatively cheaper compared to modern medicines [3,4]. Secondary metabolites play a vital role in the developmental processes of medicinal plants. Based on therapeutic behavior against various chronic or acute diseases, complex tannins, phenolic acids, steroids, flavonoids, lignans, saponins and alkaloids are considered the most effective metabolites that have been isolated and characterized from natural sources, especially medicinal plants [5,6,7]. The better adaptability and compatibility of natural products with the human body with fewer side effects and better cultural acceptability give great importance to the emergence and utilization of herbal products in the medicinal field. Plant-derived secondary metabolites with free radical scavenging activities have shown great relevance as chemo-preventive agents against free radical-associated diseases [8].

In biological systems, phenolic compounds and flavonoids may act as singlet oxygen and free radical scavengers. Phenolics are among the major phytochemicals that have been considered bioactive compounds with health benefits based on clinical trials and epidemiological studies of oxidative stress-related diseases [8]. The majority of the species in the genus Anogeissus are represented by phenolic constituents and flavonoids such as gallic acid, ellagic acid, methyl gallate, 3,4,3′-Tri-O-methylflavellagic acid, and 4,4’-(3,4-dimethyltetrahydrofuran-2,5-diyl) diphenol. The functions of saponin include its actions as an expectorant, emulsifier, and antifungal [9,10]. With the expansion of terpenoid research, it has been discovered that these molecules play an increasingly important role in the field of medicine and have a wide range of biological activities as antitumor, anti-inflammatory, antiviral, antibacterial, and antimalarial medicines, in preventing and treating cardiovascular diseases, and in exhibiting antioxidant, antiaging, immunomodulatory, and neuroprotective effects [3,11].

The rise of multidrug-resistant (MDR) pathogens is threatening the therapeutic effectiveness of several current medicines [12]. Infectious diseases induced by resistant microorganisms are linked with prolonged hospitalizations, higher costs, and an increased risk of morbidity and death [13]. The impact is seen most acutely in developing countries, where expensive replacement medications for treating highly resistant infectious illnesses are unaffordable [3]. The resistance issue necessitates renewed efforts to screen numerous medicinal plants for possible antibacterial characteristics [14].

Diabetes is another alarming issue that is prevalent these days [15]. This disease takes up a growing part of national and international healthcare budgets. From 2012 to 2014, around 1.5 million individuals died from complications of this disease. Asia and Africa have the greatest potential for suffering from this illness, with DM rates potentially increasing two- to three-fold above current levels [16].

Currently, cancer is the most complex and the deadliest disease globally. According to a report from the International Cancer Observatory, in the year 2020, 9.9 million people died due to cancer [17,18]. Conventional chemotherapy has been reported as unfavorable for cancer treatment due to its adverse side effects as well as the growing resistance towards various anticancer medications. Therefore, due to a need for treatments for various malignancies, attention is required to shift to natural sources, as many higher plants have been proven to have potent antioxidant compounds, which can lead to potent anticancer agents [19].

Natural antioxidants are broadly disseminated in medicinal plants and are responsible for a wide range of biological effects [20]. Antioxidants have been used in cancer and diabetes treatment to decrease oxidative stress, which has been linked to a reduction in long-term problems [21]. Scientists are still looking for potent, safe, and natural antioxidant medicines despite recent advances in antioxidant treatments [20].

The genus *Anogeissus* (Combretaceae) consists of around eight plant species comprised of shrubs and trees and reported for ethnomedicinal significance [22]. In India, *A. latifolia* gum is used in the form of laddu to alleviate backache and heal damaged tissue after childbirth. Some of the genus’ species are used to treat gastric disorders, skin problems, wound healing, diarrhea, diabetes, cough, dysentery, and burns. In Africa, *A. leiocarpus* is used for the treatment of malaria, helminthiasis, trypanosomiasis, diabetes, diarrhea, and wound healing [23].

A variety of bioactive metabolites has been reported from this genus, such as alkaloids, saponins, flavonoids, anthraquinones, glycosides, tannins, terpenoids, steroids, and xanthones, which are well known for their multiple health benefits and significant pharmacological activities [22]. Most of the 55 bioactive compounds that have been found in the genus are phenolic compounds [24].

*Anogeissus dhofarica* A. J. Scott (Combretaceae), genus *Anogeissus*, is the only species from this genus whose phytochemical and pharmacological behavior has not been investigated. According to geographical and botanical surveys, it has been discovered to be the most prominent tree species in the monsoon fog oasis regions of central and southern Arabia. It is a 12-m-tall tree that is sometimes reduced to a shrub by browsing and chopping [25]. With the onset of the dry season in November/December, its leaves fade from a bright green to a bluish-green hue before falling off. The crowns of the tiny, yellowish blooms are globose. *A. dhofarica* is endemic to a 300-km-long coastline strip in southeastern Yemen and the Dhofar area of southwestern Oman (both sides of the Yemeni-Omani border) [26]. This plant species is used for its foliage as fodder and is presumed to increase milk production. In Oman, women utilize soaking dried leaf water for personal hygiene, washing, and antibacterial purposes [27]. *A. dhofarica* leaves are used as a paste around infected wounds as an antibacterial and in the treatment of sores; however, specific confirmation is still required.

Therefore, the current study is conducted to gather information for the first time about the effectiveness of the chosen plant for treating various health problems and to emphasize the significance of natural products in treating such issues. In general, we aim to attract scientists by screening this plant for phytochemical composition and by performing an in vitro analysis of the antibacterial, antioxidant, and antidiabetic potential of *A. dhofarica*, which has never been investigated before. The exploration for combinations of new antioxidants and antimicrobial, cytotoxic, and antidiabetic medications among the vast sources of medicinal plants is intensifying.

## 2. Material and Methods

### 2.1. Apparatus and Reagents

The majority of the reagents were bought from Sigma-Aldrich Chemical Company (St. Louis, MO, USA). A Milli-Q (Millipore, Bedford, MA, USA) water purification system filtered deionized water. The compounds’ masses were determined using high-resolution electrospray ionization mass spectrometry (HR-ESI-MS, Agilent technologies, 6530, Q-TOF LC/MS, Agilent, country of origin USA/EU, produced in Singapore). Organic solvents were purchased from the Fisher Scientific company (Loughborough, UK). Rotavapor (BUCHI, 2017, Flawil, Switzerland) was used for the distillation and evaporation of organic solvents. A UV–visible spectrophotometer was used for absorbance.

### 2.2. Collection and Identification of Plant Samples

The whole air-dried plant of *A. dhofarica* was collected (November 2020) from Jabl-e-Taqa, Dhofar, Salalah, Oman. Dr. Syed Abudallh Gilani, a taxonomist at the Department of Biological Sciences and Chemistry at the University of Nizwa in Oman, identified the specimen using the available literature [26]. The plant specimen was photographed, and a voucher specimen (ADA/11/2020) was placed and preserved properly in the herbarium of the Natural and Medical Sciences Research Center, University of Nizwa, Sultanate of Oman.

### 2.3. Extraction and Fractionation

A dried plant sample of *A. dhofarica* (4.5 Kg) was crushed through an electric grinder into a very fine powder. A total of 4.3 Kg powder was obtained, which was then packaged and stored in the refrigerator at 4 °C until further usage. A total of 3.5 Kg of fine powder was soaked in commercial-grade methanol (5 L). After soaking for 21 days, the mixture was shaken at regular intervals and filtered through a fine cotton cloth. The residue left was again immersed in 10% water/methanol for a further 10 days with continuous shaking and filtered again. The filtrates obtained from the above-described process were merged to homogenize them, and they were then subjected to a rotary evaporator at 40 °C to permit evaporation. The resultant semi-solid mass (600 g) that had been partly evaporated was then put in the fuming hood for complete evaporation before being stored in airtight containers for later use.

**Scheme 1:**
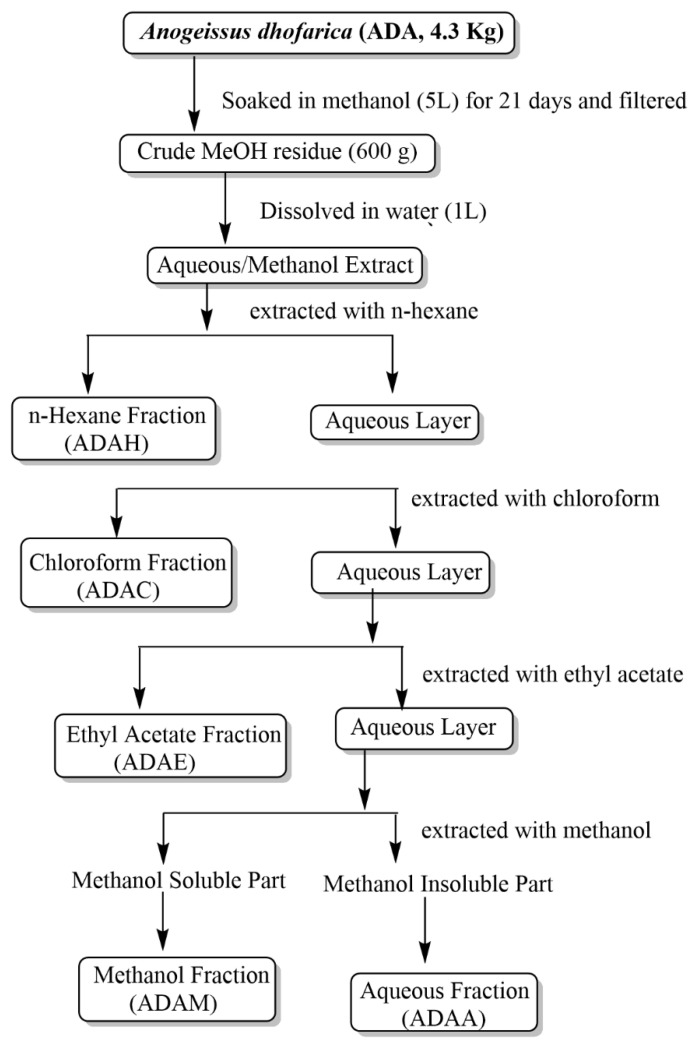
General fractionation scheme for the solvent-solvent extraction of *A. dhofarica*.

Fractionation of the crude extract was carried out by taking 500 g of methanol (ADAM) extract, first homogenized in 1 L distilled water, which was subsequently extracted using a separating funnel through solvent-solvent extraction (Scheme 1). Extraction began by shaking the crude that was immersed in distilled water with a non-polar solvent and then gradually shaking it with high-polar solvents, including n-hexane, chloroform, ethyl acetate, and aqueous solvents, to obtain four fractions with codes ADAH (31.34 g), ADAC (24.95 g), ADAE (29.42 g), and ADAA (65.32 g). Solvents from each obtained fraction were then removed using Rotavapor at a temperature of 40 °C and 120 rpm to obtain the corresponding completely dried targeted forms. The extracted % yields were calculated by the following Equation (1) and tabulated in Table 1.

(1) $$\%\;\mathrm{yield}=\frac{\mathrm{Mass}\;\mathrm{of}\;\mathrm{obtained}\;\mathrm{dry}\;\mathrm{fraction}}{\mathrm{mass}\;\mathrm{of}\;\mathrm{dry}\;\mathrm{starting}\;\mathrm{material}}\times 100$$

### 2.4. Phytochemical Analysis

Two grams of each fraction were taken and homogenized with 10 mL of dimethyl sulfoxide (DMSO) to arrange stock for numerous phytochemical and biological screening investigations. The stock was stored in a refrigerator at 4 °C.

#### 2.4.1. Qualitative Assessment

Methanol (ADAM) extract and different fractions (ADAH, ADAE, ADAC, and ADAA) of *A. dhofarica* were subjected to qualitative analysis via standard techniques to evidence the presence of various groups of compounds such as flavonoids, carbohydrates, alkaloids, and phenols [28,29,30,31].

##### Phenols

The presence of phenolic groups was identified by treating a few drops of each extract with FeCl_3_ solution in test tubes. With shaking, the appearance of bluish-green color impressions of the mixture affirmed the phenol group’s presence.

##### Flavonoids

Flavonoids were detected by treating a few drops of each extract with a 5% NaOH solution and then adding a few drops of hydrochloric acid. The disappearance of the solution color (yellow color into colorless) indicated the presence of flavonoids.

##### Carbohydrates

The presence of carbohydrates was investigated by taking 3 mL of each extract along with fractions in a glass tube with the further addition of 2 mL of Benedict reagent (research grade). Subsequently, the mixture was heated in a hot water bath for three minutes. The presence of carbohydrates was shown by the formation of reddish-brown precipitates.

##### Alkaloids

The presence of alkaloids was analyzed via Dragondroff’s reagent technique. A total of 0.5 mL of each extract was taken in a glass tube. Then, 2% sulfuric acid (H_2_SO_4_) was added, followed by heating the mixture in a hot water bath for about 3 min. After that, a few drops of Dragondroff’s reagent were added to the hot mixture. An orange-red precipitation was taken as evidence of the presence of alkaloids.

##### Saponins

The presence of saponins was confirmed by taking a few drops of stock solution of each extraction in glass tubes, followed by the addition of 0.5 mL of distilled water. The production of bubbles (froth) after heating indicated the presence of saponins in the prepared extracts.

##### Glycosides

Glycosides were revealed by the Fehling solution test. The first step was the hydrolysis of glycosides with a few drops of HCl added in test tubes with 1 mL of each extract solution. The mixture was then neutralized by adding NaOH, followed by the addition of 0.5 mL Fehling solutions (A and B). Confirmation for glycosides was revealed with a red-colored precipitation action of the solutions.

##### Tannins

The addition of a couple of magnesium ribbon fragments to 5 mL of each stock extract solution was performed, followed by the dropwise addition of concentrated HCl. The presence of a pink-crimson color revealed the presence of tannins in the extracts.

### 2.5. Phenolic and Flavonoid Content Quantification

A quantitative investigation of the total number of phenolic acids and flavonoids in the crude extract and their fractions of the selected plant was conducted using the following standard protocols.

#### 2.5.1. Quantification of Total Phenolic Content (TPC)

##### Preparation of Standard and Its Dilutions

Sánchez’s Folin–Ciocalteu approach, with some modifications, was used to determine the phenolic content in the crude methanolic extract and fractions of the chosen plant species [32]. Gallic acid was used as the reference for estimating TPC. By dissolving 0.01 g of gallic acid in 10 mL of methanol (1 mg/mL), a standard stock solution was prepared. Further different concentrated standard dilutions (1000 μL each) were prepared by taking 50, 100, 150, 200, 250, 300, 350, 400, 450 and 500 μL stock solution in ten test tubes and diluting them to 1000 μL with the addition of methanol. To each 1000 μL diluted form, 150 μL of Folin–Ciocalteu reagent (FCR) (diluted with D. water in a 1:1 ratio) and 500 μL of 7.5% Na_2_CO_3_ were added. The final volume of each dilution reached 1.650 mL. After well shaking, the prepared standard solutions of the above-described concentrations were then incubated at 37 °C for 40 min in a hot water bath to provide suitable conditions for the completion of redox reactions, which were visually observed by a change in the color of the solution to dark blue. Then, 200 μL from each standard dilution and sample were injected into the wells of a 96-well microplate. Then, through a microplate detector, the UV absorbances of the standard diluted series were recorded at 760 nm. The experiment was repeated three times. Average absorbance values were then calculated for different concentrated standard dilutions, and then a standard calibration curve was plotted between concentration and absorbance.

##### Preparation of Samples for the Estimation of Total Phenolic Contents

For sample preparation, 2 mg dry weight of each extract was taken into test tubes and dissolved into 2 mL of methanol (1000 ppm). Then, 200 μL of each methanolic extract was taken and further diluted with the addition of 800 μL of methanol. Later, the procedure of the addition of FC reagent and 7.5% Na_2_CO_3_, followed by the provision of an incubation period and the selected absorbance range, was the same as described above for standard gallic acid dilutions. Each sample was prepared in triplicate. The average of the observed absorbance values was taken to determine the total phenolic content levels in samples with units in mg gallic acid equivalents/gram sample in dry weight (mg GAE/g). The calculation of phenolic contents calculation was carried out using Equation (2).

(2) $$A=c\frac{V}{m}$$

where *A* = total phenolic content in mg GAE/g dry extract, *c* = concentration of gallic acid obtained from calibration curve in mg/mL, *V* = volume of extract in mL, and *m* = the mass of the dry extract in grams.

#### 2.5.2. Quantification of Total Flavonoid Content (TFC)

##### Preparation of Standard Quercetin for Calibration Curve

Total flavonoid contents in the extracts were determined by the aluminum chloride colorimetric assay reported by Kaur et al. [33] with some modifications. A stock solution (1 mg/mL) of quercetin was prepared by dissolving 1 mg of quercetin in 1 mL of methanol (1000 ppm). The standard solution was diluted serially to make various concentrations of 10, 20, 30, 40, 50, 60, 80 and 100 μg/mL solutions by taking volumes of 10, 20, 30, 40, 50, 60, 80, and 100 μL, respectively, from the stock solution and diluting them to 1000 μL of methanol. All the prepared solutions were transferred to the test tube. At the same time, 0.3 mL of 5% NaNO_2_ was added to the test tube, followed by the addition of 0.6 mL of 10% AlCl_3_ after 5 min. After 6 min, 0.8 mL of 1M NaOH was added to the mixture to make the total volume 2.7 mL. The above-described different concentration standard solutions were prepared in triplicate. The UV absorbances were measured at 510 nm via a microplate reader. The total flavonoid contents for each concentration was expressed as quercetin equivalents using the linear equation based on the calibration curve obtained through the average absorbance values on the y-axis and the different provided concentrations on the x-axis.

##### Preparation of Samples for Estimation of Total Flavonoid Contents

The crude extract and its fractions (1 mg) were dissolved in 1 mL of methanol to make solutions with a concentration of 1 mg/mL. Then, 200 μL from each of the prepared extract solutions was further diluted with the addition of 800 μL of methanol to give the concentration of 200 μg/mL. Later, the procedure for the addition of the required concentrations of 5% NaNO_2_, 10% AlCl_3_, and 1M NaOH with incubation periods in each interval of the addition of reagent chemicals and the selected absorbance ranges was the same as described above for the standard quercetin dilutions. Readings were taken in triplicate. Calculations for estimating total flavonoid contents in sample extracts were carried out by applying the same formula used for phenolic content determination.

### 2.6. HR-ESI-MS Analysis

The ADAM extract of *A. dhofarica* was investigated to highlight its chemical ingredients via an Agilent 1260 Infinity (Waldbronn, Germany) connected with high-resolution electrospray ionization mass spectrometry (HR-ESI-MS, Agilent technologies, 6530, Q-TOF LC/MS, Agilent, Singapore). For the Q-TOF LC/MS system, nitrogen gas was used as the nebulizer gas at 27.5 psi, 350 °C and a flow rate of 8 L/min from a nitrogen generator (PEAK scientific, Inchinnan, UK). The capillary voltage was fixed at 3.3 kV; however, the sample flow rate was kept at 10.5 μL/min. The mass range was adjusted from 30 to a maximum of 2500 *m/z*. Scan source parameters were: Vcap = 4500; nozzle voltage (V) = 2000; fragmentor = 175; skimmer1 = 6 and octopole RF peak = 750. The scan rate (spectra/sec) was 1.00. In the mobile phase, the MeOH and acetonitrile ratio was fixed at 80:20 (*v*/*v*). Mass spectra were recorded in the negative ionization mode through the standard method. Parameter sources were kept the same for all analyses.

### 2.7. Biological Activities

The screening tests of plant fractions (ADAH, ADAE, ADAC, ADAM and ADAA) were conducted to examine their potential against the microbes, and their significance as an antioxidant agent was analyzed through in vitro analysis and an assessment of their cytotoxic potential.

#### 2.7.1. Antimicrobial Activity

##### Bacterial and Fungal Culture

All the organisms used in this study were obtained from the microbiology laboratory, NMSRC, University of Nizwa, Oman. Isolates of the following bacteria and fungi were used in this experiment: (*Staphylococcus aureus* (ATCC 29213), *Enterobacter hormaechei* (ATCC 700323), *Candida albicans* (ATCC 14053) and *Candida kruzei* (ATCC 6258).

##### Preparation of Bacterial Inoculum

Bacterial strains were inoculated on nutrient agar (Liofilchem, Teramo, Italy) and incubated for 24 h at 28 °C. The inoculum was prepared using the direct colony suspension method. Three to five morphologically similar colonies were transferred with a loop from fresh nutrient agar into about 5 mL of normal saline in a capped test tube and vortexed. The suspension was adjusted to have the turbidity of a 0.5 McFarland standard. At the end of incubation, the colonies were counted and expressed as colony-forming units per milliliter (1.5 × 10^8^ CFU/mL).

##### Preparation of Fungal Inoculum

Freshly subcultured fungal strains were incubated on sterile potato dextrose agar (PDA) (Liofilchem, Italy) for 24–48 h at 28 °C. The resulting cells were washed in sterile normal saline, and their turbidity was accustomed to a McFarland standard equivalent of 0.5, yielding 1 × 10^6^ CFU/mL colonies.

##### Antibacterial Assessment

The agar diffusion procedure was used to assess the antibacterial activity [34]. Each extract of 0.1 g was homogenized in 1 mL of dimethyl sulfoxide (DMSO) to obtain a 1000 ppm solution, from which 50 μL from each homogenized solution was taken for further analysis. The suspension of 0.5 McFarland was inoculated on Muller–Hinton agar (MHA) plates (Liofilchem, Italy) using a cotton swap in a continuous zigzag manner. The medium was punched via a cork-borer, and 50 μL of the corresponding material was added. Punches were made for subjecting the standard and the blank (DMSO). Both were incubated at 28 °C for 24 h; then, results were recorded by measuring the zone of inhibition around the discs in mmL. *S. aureus* (ATCC 29213, Gram-positive bacteria) and *E. hormaechei* (ATCC 700323, Gram-negative bacteria) were used to test the antibacterial activity of *A. dhofarica* extracts. Ciprofloxacin was used as a standard for the *S. aureus* strain, while gentamicin was used for the *E. hormaechei* strain.

##### Antifungal Assessment

The antifungal activity of each extract (ADAH, ADAE, ADAC, ADAM, and ADAA) against *Candida albicans* (ATCC 14053) and *Candida kruzei* was also evaluated (ATCC 6258). Amphotericin was used as a standard for *C. albicans and C. kruzei*. Using the diffusion technique, the antibacterial activity of plant extracts was evaluated. Residues of plant extract were dissolved separately in DMSO with a maintained final concentration of 0.1 g/mL. Modified Mueller–Hilton agar (MHA), 2% glucose and 5 µg of methylene blue/mL were poured into Petri dishes. Each punch was loaded with 50 µL of microbial suspension. A filter paper disc containing 5 mg of amphotericin and a filter paper disc simply containing the solvent (without plant extract) were put above modified MHA plates as positive and negative controls, respectively. All Petri plates were maintained at room temperature for one hour to allow the plant extract to diffuse and then incubated at 28 °C for 24 to 48 h. Incubation results were monitored.

##### Minimum Inhibitory Concentration Determination

To determine the minimum inhibitory concentration (MIC) of the crude extracts of *A. dhofarica,* serial dilutions from a stock of 1000 ppm solution of each fraction were made. From the stock solution of 0.1 g/mL, further dilutions with a 2-fold dilution factor were prepared (1/2, 1/4, 1/8, 1/16, 1/32, 1/64, 1/128, 1/256 and 1/512) equal to 50.0, 25.0, 12.50, 6.25, 3.125, 1.5625, 0.78125, 0.390625 and 0.1953125 mg/mL. Plates were inoculated by microorganisms, and several wells were made on each plate and filled with 50 μL of solutions. Plates were incubated at 28 °C for 24 h for the bacteria and 48 h for the yeasts. The sensitivity of the test microorganisms to the crude extracts and the standard antibiotics were indicated by clear zone around wells and discs. The inhibition was measured with a ruler and expressed as the degree of sensitivity after measuring the diameter of inhibitory zones formed around each hole in mm. The calculation was carried out using Equation (3) to obtain the result of the MIC in mg/mL for the last concentration that appeared with a zone of inhibition.

MIC = D × 0.1 g/mL = g/mL × 10_3_ = mg/mL (3)

where D is the dilution value, and 0.1 g/mL is the starting concentration (stock).

#### 2.7.2. Antioxidant Activity

The antioxidant activity of the extracts was assessed based on the radical-scavenging effect of the stable 1,1- diphenyl-2-picrylhydrazyl (DPPH 0.004%) free radical. The methodology applied was reported by Kedare et al. [35]. DPPH (4 mg) was homogenized properly in 100 mL of distilled methanol to prepare 0.004% DPPH solution. The prepared solution was placed in the dark for 30 min in the sense of the formation of free radicals in the solution. The methanolic extracts (1 mg/mL) were taken in glass tubes in different concentrations (1000, 500, 250, 125 and 62.5 μg/mL), and then each concentration was further diluted by making up to 3ml with methanol. To take readings through the microplate reader, only 150 μL of different concentrations of extracts was taken, followed by the addition of 150 μL DPPH. This mixture was then placed in the dark at room temperature for 30 min. The samples with the blank (only methanol) and control (methanol + DPPH solution) were taken in a 96-well microliter plate. A decrease in absorption for each solution was measured at 517 nm using a microplate reader. All experiments were repeated three times, and the average value was taken as the result. Ascorbic acid was used as a reference. The percentage of the DPPH radical scavenging was calculated using Equation (4):

(4) $$\%\;Free\;radicals\;scavenging\;activity=\frac{Acontrol\;–\;Bsample\;}{A\;control}\times 100$$

where *A_control_* represents the absorbance of the control and *B_sample_*_:_ denotes the absorbance of the sample.

#### 2.7.3. α-Glucosidase Assay

Crude extracts were tested in vitro against the -glucosidase enzyme (EC 3.2.1.20) using the Shah et al. approach [36] and using a 50 mM phosphate buffer of pH 7. (6.8). The enzyme (1U/2 mL) was adequately dissolved in phosphate buffer at a volume of 20 μL/well for the enzyme and 135 μL/well for the phosphate buffer. A total of 20 μL of each sample was dissolved in DMSO (μg/mL) and placed in 96-well plates incubated at 37 °C for 15 min. After the incubation period, the substrate para-nitrophenyl-*α* D-glucopyranoside with a concentration of 0.7 mM (25 μL) was added in each, and the change in absorbance was observed via a microplate reader at 400 nm for a time period of 30 min. Firstly, screening was done for a concentration of 500 μg/mL of each extract; then, the IC50 was evaluated for those who appeared active against the enzyme by taking two-fold concentrations (dilutions) ranging from 10, 100, 250, and 500 μg/mL. The positive control acarbose with an IC_50_ of 377.0 ± 1.06 μg/mL was used, and DMSO was used as a negative control. The % inhibition was calculated using the following Equation (5):

(5) $$\;\;\;\;\;\;\%\;Inhibition=100-\left(\frac{OD\;test\;sample}{OD\;control}\right)\times 100.\;\;\;\;$$

where *OD _test sample_* represents the optical density of the sample and *OD _control_* represents the optical density of the control.

#### 2.7.4. Cytotoxicity Assay

The MTT (yellow tetrazolium salt, 3-(4,5-dimethylthizol-2-yl)-2,5-diphenyl tetrazolium bromide) test was used to assess the in vitro cytotoxicity of metal complexes with the aggressive breast cancer cell line MDA-MB-231. This line serves as an excellent model of triple-negative breast cancer and is commonly used for in vitro studies. Human breast normal cell line MCF-10A was kept as a control in this study. Cells were cultured in DMEM supplemented with 10% FBS and 1% antibiotics (100 U/mL penicillin). At a density of 1.0 104 cells per well, the cells were seeded in a 96-well plate and incubated for 24 h at 37 °C with 5% CO_2_. After discarding the media, various concentrations of plant extracts (1.25, 2.5, 5, and 10 mg/mL) were applied to both cell lines [37,38]. Following 48 h of incubation, 20 μL of MTT solution (5 mg/mL) was pipetted into each well and then incubated for an additional 4 h. The medium was later discarded, and the formazan precipitate was dissolved in DMSO. The absorbance of the mixtures was determined using a microplate reader at 570 nm. All experiments were carried out in triplicate, and cytotoxicity was determined as a percentage of cell viability relative to untreated control cells (Equation (6)).

(6) $$\%\;Viability=\frac{Absorbance\;of\;sample}{Absorbance\;of\;control}\times 100$$

#### 2.7.5. Cytotoxic Assay (Brine Shrimp Lethality Assay)

A brine shrimp lethality assay was performed to determine the cytotoxic effect of each prepared extract of *A. dhofarica* via brine shrimp (sea monkeys) assay, where brown-colored, 1 mm-sized invertebrate shrimp organisms were used.

##### Shrimp Larvae Hatching

The cysts (eggs) of 50 g of *Artemia salina* were taken into artificial seawater (38 g sea salt in 1 L distilled water) and transferred into a plastic container, which was divided into two compartments. To provide a dark zone for hatching the shrimp larvae, one of the container compartments was covered with aluminum foil, while the other was illuminated. After a period of 24 h incubation, the mature larvae (nauplii) were attracted toward the lightened zone and were then collected with a pipette [39].

##### Brine Shrimp Lethality Assay

Selected plant extracts’ toxicity potential was tested by evaluating their cytotoxic activity. The evaluation methodology was well described by Al-matani et al. [39] and Afaf et al. [40]. Two-fold dilutions were prepared for all five extracts dissolved in DMSO (1 mg/mL) with concentrations of 10, 100, 250 and 500 μg/mL. Each concentration (100 μL) was injected into appropriately labeled glass tubes, and the volume was increased by adding 5 mL of artificial seawater. Potassium permanganate was taken as a positive control. We proceeded with a similar method of preparing various concentrations as described above for the dilution of the extracts. Then, 10 nauplii were added to each of the glass tubes. DMSO was taken as a negative control, having no sample. The vials were kept at room temperature for 24 h. After that period, the number of dead nauplii was counted, and mortality was calculated using Equation (7).

(7) $$Mortality\;\%=\frac{Total\;No.-Survived\;No.}{Total\;No.}\times 100\;$$

### 2.8. Statistical Analysis

One-way and two-way analysis of variance (ANOVA) were performed using Bonferroni’s test (*p* ≤ 0.05) to determine the statistical significance level of the quantitative results. The significance of antioxidants was assessed using a nonlinear regression graph (NLRG) that displayed concentration and percent inhibition, and the IC50 was determined using Equation (7) utilizing the GraphPad Prism 9 application for Windows (GraphPad-Software 9.5.0, San Diego, CA, USA, 2020).

IBM SPSS Statistics 26 software was used to evaluate the dose-response and computation IC_50_ values for the in vitro cytotoxic assay. The LC_50_ for brine shrimp mortality was measured through a nonlinear Equation (8) obtained from statistical data using Microsoft Excel.

(8) $$\;Y=100/I+\left(ˆHill\;Slope\right)\;$$

where *I* represents the inhibitors’ concentration, *Y* represents the inhibitor’s reaction, and hill slope represents the curve’s steepness.

## 3. Results and Discussion

*A. dhofarica* was selected to perform a complete phytochemical profiling and in vitro biological investigation. For this purpose, its crude extract (ADAM) and sub-fractions (ADAH, ADAC, ADAE, and ADAA) were prepared and investigated for their phytochemical profiling through the HR-ESI-MS technique, and its in vitro antibacterial, antifungal, cytotoxicity, breast cancer, antidiabetic and antioxidant potentials were assessed using standard protocols with slight modifications. The need for an HR-ESI-MS study lay in the fact that the plants can produce secondary metabolites gradually according to the stress created due to environmental factors such as temperature, soil fertility, soil water, and salinity. These factors have a significant impact on some of the processes happening in plant bodies that result in significant alternations in entire phytochemical profiles and make them compete potentially against unfavorable conditions [41]. As this plant species has not been previously investigated regarding these above-described phytochemical and biological parameters, this study was conducted to support the beneficial usage of the selected plant and provide the scientific validation of *A. dhofarica* in terms of its phytochemical composition and observed biological potentials.

### 3.1. Qualitative Phytochemical Analysis

To highlight the significance of *A. dhofarica,* the phytochemical identification of its representative groups was performed. Based on the detection of significant chemical entities, the crude extract of the selected plant and their fractions further proceeded to various biological studies. Investigation revealed that flavonoids, phenols, carbohydrates, tannins, and glycosides were found in all tested samples, showing positive results for all applied tests except alkaloids. The absence of alkaloids from the same genus species was also reported by Adeleye et al. [34] and Mann et al. [34,42]. A comparative study of crude extracts revealed that the n-hexane fraction showed very little or no detection of flavonoids, carbohydrates, tannins, and glycosides. Flavonoid-type compounds were majorly found in the extracts compared to other classical compounds. The flavonoids dominating the findings were totally inconsistent with the data provided in the literature regarding this genus. The obtained results for the presence of phenols were in complete agreement with the data reported by Konate et al. [43] for *A. leiocarpus*. The results are summarized in Table 2 for quick interpretation.

### 3.2. Total Phenolic and Flavonoid Contents

The estimation of total phenols as well as flavonoid contents in the extracts of *A. dhofarica* plant species are shown in Table 3 and Tables S1 and S2. The quantification of total phenolic contents (TPC) is reported as mg Gallic acid equivalent/g of the extract with a standard curve reference (y = 0.0018x + 0.3515, R^2^ = 0.9885) (Figure S1). Contents varied between fractions, and some were shown to be significant, as demonstrated by a *p*-value < 0.05. TPC ranged from 83.14 ± 0.5 mg GAE/g to 420.4 ± 1.9 mg GAE/g. The ethyl acetate fraction had higher phenol contents (420.4 ± 0.3 mg GAE/g), followed by the methanol fraction and the aqueous fraction, with phenolic quantities of 299.9 ± 0.5 mg GAE/g and 210.8 ± 1.2 mg GAE/g, respectively, compared to the chloroform fraction (148.2 ± 0.5 mg GAE/g). The least quantity of phenolic contents was found in the n-hexane fraction (83.14 ± 0.5 mg GAE/g). The obtained results for phenolic contents correlated with the results reported by Olugbami et al. and Elufioye et al. [8,44] for *A. leiocarpus* and Danai et al. [45] for *A. pendula*.

The results of the flavonoid contents of extracts were reported as (mg QE/g) of the extract with a standard curve reference (y = 0.0018x + 0.5734, R^2^ = 0.9785) (Figure S2) and were present in a range from 34.4 ± 0.4 mg QE/g to 94.1 ± 0.3 mg QE/g, with higher flavonoid quantities in methanol (94.1 ± 0.3 mg QE/g), followed by ethyl acetate (80.2 ± 0.1 mg QE/g). The aqueous and chloroform fractions contained 69.6 ± 0.2 mg QE/g and 56.2 ± 0.3 mg QE/g of flavonoids, respectively, while the least quantity was observed in the n-hexane fraction (34.4 ± 0.4 mg QE/g). Flavonoid contents for the *A. dhofarica* plant extracts correlated with the quantitative estimation data reported by Olugbami et al. [8] and Annegowda et al. [46] for *A. leiocarpus* and *T. catappa,* respectively. The polarity of the solvents significantly affected the distribution of phenolic and flavonoid contents [3]. The high TPC and TFC values of *A. dhofarica* fractions should be a means of predicting their potential therapeutic relevance because phenolics and flavonoids have been linked with various biological properties [47].

### 3.3. HR-ESI-MS Assessment

**Figure 1:**
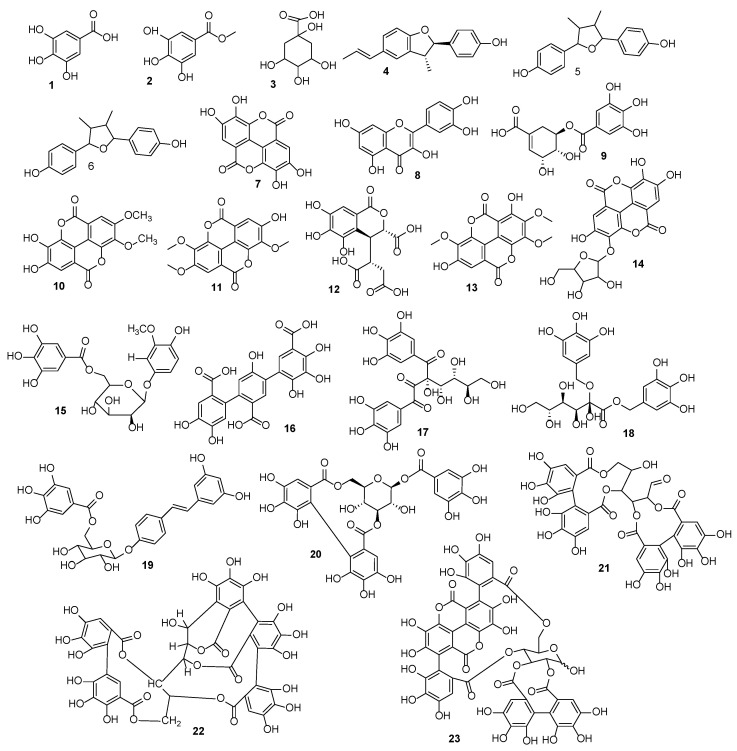
Structures of the tentatively identified compounds in ethyl acetate (ADAE) and methanol (ADAM) extracts of A. dhofarica.

Based on the most substantial abilities for the observed biological activities, the most active extracts (ADAM and ADAE) were profiled to tentatively identify their promising bioactive ingredients. The tested samples contained 22 compounds (Figures S3–S6), including polyphenols consisting of two major classes, flavonoids and non-flavonoids, which were further identified to the level of sub-classes as five flavanones and one flavonol. The tested sample also contained non-flavonoid subclasses, including four phenolic acids, three lignans, four ellagitannins, four gallo-tannins, one gallic ester and one aliphatic carboxylic acid. All the chemical entities were detected in negative ionization mode (NIM). The full scan chromatograms of the negative ionization mode and the chromatograms of the identified compounds are given in the Supplementary Materials. Flavanones were noted as a major group and contributed five compounds (**7**, **10**, **11**, **13** and **14**), which were identified in negative ionization mode (NIM); they are displayed in Table 4 and Figure 1. These compounds were reported for the first time in *A. latifolia* [48] and later reported in *A. leiocarpus* [49], *A. acuminata* [50], *A. schimperi* [51], and *A. pendula* [52]. They possess the promising potential to resist human pathogenic microbes, scavenge free radicals and be cytotoxically significant [53]. In addition, four gallo-tannins (**15**, **17**, **18** and **19**) and four ellagitannins (**20**, **21**, **22** and **23**) were observed in NIM in the most active extract, having the therapeutic potential to serve as an antioxidant, antimicrobial, and anti-diabetic agent [53,54].

### 3.4. Antimicrobial Potential

#### 3.4.1. Antibacterial and Antifungal Assessment

The antimicrobial potential of *A. dhofarica* extracts was tested against Gram-negative bacteria (*E. hormaechei*), Gram-positive bacteria (*S. aureus*), and two fungal strains (*C. albicans* and *C. kruzei*), as well as standards and blanks (Table 5). In the case of Gram-positive bacterial strains, the ADAE fraction demonstrated the highest resistance against *S. aureus* with a ZOI of 28 ± 0.3 mm, followed by ADAA (26 ± 0.3 mm) and ADAM (26 ± 0.2 mm). In comparison to ADAC (ZOI, 16 ± 0.1 mm), the ADAH fraction had a slightly higher inhibition of 17 ± 0.3 mm. None of the fractions inhibited the growth of the Gram-negative *E. hormaechei*. The findings were compared to those of Sani et al. and Govindarajan et al. [61,62]. No activity against Gram-negative bacteria could be justified because Gram-negative strains have significantly less peptidoglycan content in their cell wall than Gram-positive strains, resulting in high drug resistance [61,63]. In terms of inhibition against fungal strains (yeasts), the extracts produced significant inhibitory results for *C. kruzei* growth inhibition, while *C. albicans* inhibition potential was comparatively unsatisfactory. The ADAA fraction was found to be the most active participant in both fungal strains, with 34.0 ± 0.1 mm and 18.02 ± 0.2 mm zones of inhibition for *C. kruzei* and *C. albicans*, respectively. The inhibitory results for the ADAM fraction were slightly different from the ADAA results, with ZOI 30.01 ± 0.2 mm and 16.02 ± 0.2 mm for *C. kruzei* and *C. albicans*, respectively, indicating that the ADAA and ADAM fractions have similar potential against fungal strains, which could be due to the presence of similar polar phytochemical composition in both fractions. For both fungal strains with ZOI 16.01 ± 0.3 mm and 9.02 ± 0.2 mm, the ADAH fraction showed the least inhibitory results. The obtained results were completely consistent with the data reported by Batawila et al. [64].

#### 3.4.2. Antimicrobial MIC Evaluation

The minimum inhibitory concentration (MIC) of *A. dhofarica* fractions for the provided bacterial strains was 0.78–3.12 mg/mL, while the MIC for fungal strain fractions was 0.78–50 mg/mL (Table 6). The MIC data range observed for extracts on *S. aureus* is completely consistent with the MIC data reported by Issa et al. [65] for the same genus species *Terminalia avicenniodes*, who also justified that the solvent system used for extraction had a significant effect on the phytochemical components exhibited by the *T. avicenniodes* extracts, which would affect the potential of the plant fractions against bacterial isolates. Our antifungal results are consistent with those reported by Batawila et al. and Baba-Moussa et al. [64,66] from other West African *Combretaceae* spp., which showed the best antifungal activity for *Terminalia avicenniodes* extracts with MIC ranges of >4 mg/mL on *Candida albicans*. According to Tona et al. [67], a chemical analysis of different solvent fractions from *A. leiocarpus*, *T. macroptera*, *C. fragrans*, and *T. laxiflora* revealed the presence of high amounts of saponins, flavonoids, and tannins, which may be responsible for their antifungal activity.

### 3.5. Antioxidant Assessment

The free radical scavenging potential was assessed for extracts of *A. dhofarica* through a DDPH test (Table 7). The ethyl acetate fraction (ADAE) contributed excellent antioxidant capacity, even superior to ascorbic acid, which was taken as a standard, with an IC_50_ = 9.8 ± 1.2 μg/mL, while the standard demonstrated an IC_50_ = 13.8 ± 0.4 μg/mL. The increased antioxidant potential of the ethyl acetate fraction over standard ascorbic acid was also reported by Aku et al. [68] for a species of the same genus, *A. leiocarpus*. The methanol extract (ADAM) and aqueous extract (ADAA) also demonstrated higher IC_50_ values of 17.4 ± 0.4 μg/mL and 25.8 ± 0.1 μg/mL, respectively, which were comparatively lower than the ADAE’s IC_50_ range. This was followed by the chloroform extract (ADAC) with an IC_50_ = 37.8 ± 0.2 μg/mL, followed by the hexane extract, which was relatively the least active in the DPPH assay, with an IC_50_ = 104.9 ± 0.3 μg/mL. According to Kodama et al. [69], plants with high phenol and flavonoid contents have significant antioxidant potential. These results are in complete agreement with the polyphenol contents found in the extracts (ethyl acetate > methanol > aqueous > chloroform > hexane). As a result, the antioxidant capacity seen in the DPPH experiment may be attributed to the presence of these compounds. Current observations reveal that our findings are completely matched and already well justified by Zadra et al. and Ragvan Govinadaraj et al. [70,71] for *S. guaraniticum* and *A. latifolia*, respectively.

### 3.6. In-Vitro Antidiabetic Assay

The extracts from *A. dhofarica* displayed potent inhibitory potential. Of all extracts, the methanol (ADAM) fraction with an IC_50_ value of 2.1 ± 0.05 μg/mL was demonstrated as the most potent antidiabetic compared with standard acarbose IC_50_ = 377.00 ± 1.06 μg/mL and the other fractions. All fractions demonstrated higher antidiabetic potential than the standard (Table 8). When comparing the extracts, a relatively low antidiabetic potential can be seen in the chloroform (ADAC) fraction, with an IC_50_ value of 20.11 ± 0.42 μg/mL. Our data somewhat contradict the data reported by Stephen et al. for *A. leiocarpus* [72]. These variations might be due to the constituent variations and approaches used among plant species.

### 3.7. Cytotoxic Activity

Different concentrations of the plant extracts in mg/mL (1.25, 2.5, 5, and 10) were used to investigate against the human breast cancer cell line MDA-MB-231 to identify their potential to inhibit the growth of cancer cells. At the same time, the MCF-10A human normal breast epithelial cell line served as a control in the experiment. An MTT (3-(4,5-dimethylthiazol-2-yl)-2,5-diphenyltetrazolium bromide) assay was assessed to determine the decrease in cancer cell viability induced by cytotoxic agents. The IC50 values, the percentage of inhibition, and the viability of plant extracts for MDA-MB-231 are shown in Table 9.

Results of the MTT assay revealed that the ADAM, ADAE, ADAH and ADAC extracts showed potent activity against MDA-MB-231 cells. Among all extracts, ADAE showed more potency toward the MDA-MB-231 cell line with an IC_50_ value of 6.2 ± 0.3 mg/mL. Our findings are in correlation with data reported by Hassana et al. [73] for the leaf extract of *A. leiocarpus*.

The non-cancerous MCF-10A cells were treated with the extracts at varying doses in the same manner as the cancer cells were to test whether the cytotoxic effects of the compounds were specific to cancer cells. The percentage of MCF-10A cell lines inhibited and the cell viability after treatment with the plant extracts were determined using the MTT test and are shown in Table 10. The findings demonstrated that these cells were less affected by the extracts’ effects, particularly the ADAE extract, which represented the greatest percentage of inhibition in breast cancer cells. According to the findings of this research, triple-negative MDA-MB-231 cells, which have an aggressive phenotype, reacted more to most plant extracts and showed greater cytotoxicity.

When non-tumorigenic MCF-10A cells were exposed to various plant extracts, their cytotoxicity was decreased. This suggests that these novel extracts have the potential to offer promising therapy for patients with breast cancer.

### 3.8. Brine Shrimp Lethality Assay (Cytotoxic activity)

**Figure 2:**
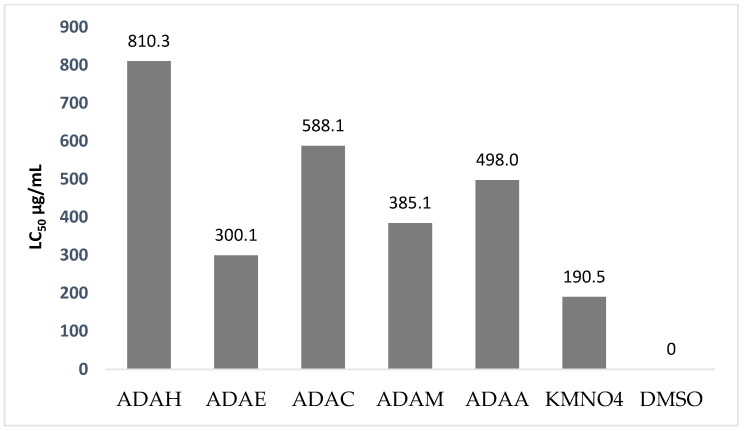
Comparison of LC50 values of different polarity extracts of A. dhofarica with positive (KMNO4) and negative (DMSO) controls.

The cytotoxic activity results were shown to be significant among the five prepared extracts (of different polarities). The percent mortality of nauplii is shown in Table 11. The order of activity was ADAE > ADAM > ADAA > ADAC > ADAH. Moreover, increases in concentration resulted in an increase in the mortality rate. Compared to the positive control (potassium permanganate, KMNO_4_), the least LC_50_ was observed for the ADAE extract, followed by the ADAM extract with LC_50_ values of 300.1 μg/mL 385.1 μg/mL, respectively (Figure 2), while the highest LC_50_ of 810.3 μg/mL was observed in the ADAH fraction. As high values of LC_50_ indicate less toxicity, it was identified that the ADAH fraction consisted of a less toxic chemical composition. In our current study, the ADAE extract was demonstrated to be highly toxic because it showed the highest LC_50_ value, which revealed the presence of potent toxic chemical compounds in its chemical composition. Our results are well correlated with the results for cytotoxicity reported by Weli et al. [40].

## 4. Conclusions

Based on the findings, it is determined that *A. dhofarica* includes responsible bioactive compounds, including a variety of phytochemicals with a wide range of biological activities, which may be responsible for its many therapeutic benefits. All the studied samples, including the ADAE extract, had the greatest levels of flavonoid and phenolic contents. A total of twenty-two bioactive compounds were identified for the first time in *A. dhofarica*, with flavonoids and phenolic acids dominating the list. ADAE and ADAM demonstrated significant antibacterial efficacy against bacterial and fungal pathogens. The ADAE extract was demonstrated to have potent DPPH free radical scavenging activities, whereas the ADAM and ADAA extracts were found to be effective against diabetes. In the in vitro cytotoxicity investigation, the ADAE extract was proven to be highly anticytotoxic against brine shrimp larvae as well as against breast cancer cell lines. As a result, it was determined that *A. Dhofarica* has the potential to be used to resist microorganisms, scavenge free radicals, cure diabetes, and fight cancer. These properties are attributed to the presence of tannins, flavonoids and phenolic acids. Still, further research is needed to screen and identify the probable chemical constituents for the complications under consideration.

## Figures and Tables

**Table 1:** Extraction efficiency in % yield.

Fractions	Obtained Mass (g)	Yield (%)
ADAH	31.34	6.26
ADAC	24.95	4.99
ADAE	29.42	5.88
ADAA	65.32	13.06

ADA, *A. dhofarica*; C, chloroform; H, n-hexane; E, EtOAc; M, methanol; A, aqueous.

**Table 2:** Qualitative phytochemical analysis of extracts of *A. dhofarica*.

Fractions	Phenols	Flavonoids	Carbohydrates	Tannins	Alkaloids	Saponins	Glycosides
ADAC	+	+	+	+	–	+	–
ADAH	+	–	–	–	–	–	–
ADAE	+	+	+	+	–	+	+
ADAM	+	+	+	+	–	+	–
ADAA	–	–	+	+	–	–	+

ADA, *A. dhofarica*; C, chloroform; H, n-hexane; E, EtOAc; M, methanol; A, aqueous. (+) = identified representative groups. (–) = not identified phytochemical groups.

**Table 3:** Flavonoid and phenolic contents in *A. dhofarica* extracts.

Extracts	Total Flavonoid Contents (mg QE/g) of Dry Fractions Mean ± SEM	Total Phenolic Contents (mg GAE/g) of Dry Fractions Mean ± SEM
ADAH	34.4 ± 0.4	83.14 ± 0.5
ADAE	80.2 ± 0.1	420.4 ± 1.9
ADAC	56.2 ± 0.3	148.2 ± 0.5
ADAM	94.1 ± 0.3	299.9 ± 0.5
ADAA	69.6 ± 0.2	210.8 ± 1.2

ADA, *A. dhofarica*; H, n-hexane; E, ethyl acetate; C, chloroform; M, methanol; A, aqueous; TFC, total flavonoid contents; TPC, total phenolic contents; GAE, gallic acid equivalent; QE, quercetin equivalent; Data expression, mean ± SEM (standard error mean), Bonferroni’s test, *n*: 3, *p* < 0.05 (results are significant); mg: milligram, g: gram.

**Table 4:** Compounds identified through HR-ESI-MS analysis in the active extracts of *A. dhofarica*.

S. No	Name	Molecular Formula	M-H Calculated	M-H Observed	Reference Species	Classification
1	Gallic acid	C_7_H_12_O_6_	169.0137	169.0115	*A. latifolia* [48]	Phenolic acid
2	Methyl gallate	C_8_H_8_O_5_	183.0262	183.0293	*A. latifolia* [48]	Phenolic acid
3	Quinic acid	C_7_H_12_O_6_	191.0555	191.0510	*A. leiocarpus* [49]	Aliphatic carboxylic acid
4	Conocarpan	C_18_H_8_O_2_	265.1225	265.1387	*A. acuminate* [50]	Lignan
5	Anolignan B	C_18_H_8_O_2_	265.1225	265.1387	*A. acuminate* [50]	Lignan
6	4,4’-(3,4-dimethyltetrahydrofuran-2,5-diyl) diphenol	C_18_H_20_O_3_	283.1334	283.0493	*A. Acuminate* [50]	Lignan
7	Ellagic acid	C_14_H_6_O_8_	300.9984	300.9893	*A. letifolia* [48]	Flavanone
8	Quercetin		301.0348	301.0241	*A. letifolia* [48]	Flavonol
9	5-O-Galloylshikimic acid	C_14_H_14_O_9_	325.0559	325.1695	*A. letifolia* [55]	Phenolic acid
10	2,3-Di-O-methylellagic acid	C_16_H_10_O_8_	329.0297	329.0193	*A. letifolia* [48]	Flavanone
11	3,4,3′-Tri-O-methylellagic acid	C_17_H_12_O_8_	343.0453	343.1137	*A. schimperi* [51]	Flavanone
12	Chebulic acid	C_14_H_12_O_11_	355.0301	355.3078	*A. leiocarpus* [56]	Phenolic acid
13	3,4,3′-Tri-O-methylflavellagic acid	C_17_H_12_O_9_	359.0403	359.0235	*A. Letifolia* [48]	Flavanone
14	Ellagic acid pentoside	C_19_H_14_O_12_	433.0407	433.2132	*A. leiocarpus* [57]	Flavanone
15	3-Methoxy-4-hydroxyphenol-1-O-𝛽-D-(6′-O-galloyl)-glucoside	C_20_H_22_O_12_	453.1039	453.0877	*A. leiocarpus* [58]	Gallotannin
16	(+)-gallocatechin-3-O-gallate	C_22_H_18_O_11_	457.0776	457.3076	*A. pendula* [52]	Catechin allates
17	Digalloylglucose	C_20_H_20_O_14_	483.0774	483.0627	A. *leiocarpus* [58]	Gallotannin
18	Digalloxyglucose	C_20_H_24_O_14_	487.1087	487.3117	A. *leiocarpus* [58]	Gallotannin
19	Resveratrol-4’-O-(6″-galloyl) glucoside	C_27_H_25_O_12_	541.1346	541.0059	*A. latifolia* [59]	Gallotannin
20	Corillagin	C_27_H_22_O_18_	633.0727	633.0506	*A. latifolia* [59]	Ellagitannin
21	Bis-HHDP glucose	C_34_H_23_O_22_	783.0681	783.3675	*A. pendula* [52]	Ellagitannin
22	Castalagin	C_41_H_26_O_26_	933.0634	933.0250	*A. acuminate* [60]	Ellagitannin glycoside
23	Punicalagin	C_48_H_28_O_30_	1083.0590	1083.0131	*A. leiocarpus* [58]	Ellagitannin

**Table 5:** Zones of inhibition (ZOI) in mm ± SEM of A. dhofarica extract and fractions against three microbial strains (*S. auris*, *C. albicans* and *C. kruzei*).

Extracts	ZOI (mm ± SEM).		
Sample Codes	Bacteria	Fungi	
	*S. aureus*	*C. albicans*	*C. kruzei*
ADAH	17 ± 0.3	NA	NA
ADAE	28 ± 0.4	14 ± 0.5	26 ± 0.1
ADAC	16 ± 0.1	NA	NA
ADAM	26 ± 0.2	16 ± 0.2	30 ± 0.2
ADAA	26 ± 0.3	18 ± 0.2	34 ± 0.1
DMSO (Blank)	NA	NA	NA
Standards	31.6 ± 0.3	16.1 ± 0.1	20.5 ± 0.4

ADA, *A. dhofarica*; H, n-hexane; E, ethyl acetate; C, chloroform; M, methanol; A, aqueous; ZOI, zone of inhibition. Standards: Ciprofloxacin for *S. aureus*, Gentamicin for *E. hormaechei*, *S. aureus* (Gram-positive), *E. hormaechei* (Gram-negative), Amphotericin B (AMB) for *C. albicans* and *C. kruzei*; DMSO, blank; NA = not active; mean ± SEM (data were taken as mean ± SEM).

**Table 6:** The minimal inhibitory concentration (MIC) of extracts required to inhibit microbial growth (mg/mL).

Extracts	MIC (mg/mL)		
Sample Codes	Bacteria	Fungi	
	*S. aureus*	*C. albicans*	*C. kruzei*
ADAH	3.12 ± 0.5	-	-
ADAE	0.78 ± 0.3	50 ± 0.5	3.12 ± 0.3
ADAC	3.12 ± 0.2	-	-
ADAM	1.56 ± 0.1	25 ± 0.1	3.12 ± 0.5
ADAA	1.56 ± 0.4	25 ± 0.1	0.78 ± 0.2

ADA, *A. dhofarica*; H, n-hexane; E, ethyl acetate; C, chloroform; M, methanol; A, aqueous; MIC, minimum inhibitory concentration; *S. aureus* (Gram-positive), *E. hormaechei* (Gram-negative), SEM, standard error of the mean; (-): not tested because plant extracts were not active by screening disc diffusion assay.

**Table 7:** Percent inhibition of free radicals (DPPH) and IC50 of extracts in (μg/mL) ± SEM of *A. dhofarica*.

Extracts	Conc. (μg/mL)	% Inhibition	IC_50_ Values (μg/mL) ± SEM
ADAH	1000	92.72	
	500	87.15	
	250	76.32	37.8 ± 0.2
	125	66.17	
	62.5	55.15	
ADAC	1000	88.92	
	500	83.75	
	250	69.811	104.9 ± 0.3
	125	54.17	
	62.5	42.97	
ADAE	1000	96.2	
	500	93.95	
	250	87.75	9.8 ± 1.2
	125	79.24	
	62.5	70.60	
ADAA	1000	95.12	
	500	91.825	
	250	85.75	25.8 ± 0.1
	125	74.454	
	62.5	65.80	
ADAM	1000	97.54	
	500	93.32	
	250	86.45	17.4 ± 0.4
	125	76.60	
	62.5	67.60	
	1000	96.12	
	500	94.02	
Ascorbic Acid	250	89.61	13.8 ± 0.4
(Standard)	125	80.14	
	62.5	68.60	

ADA, *A. dhofarica*; M, methanol; H, n-hexane; C, chloroform; Aq, aqueous. Ascorbic acid = positive control. *n*: 3. Results are significant at *p* < 0.05 (Bonferroni’s test). Data were taken as mean ± SEM.

**Table 8:** α-glucosidase activity of different polarity extracts of *A. dhofarica*.

Extracts	% Inhibition	IC_50_ ± μg/mL (SEM)
ADAH	94.30	10.1 ± 0.31
ADAE	94.86	6.4 ± 0.12
ADAC	91.21	20.1 ± 0.42
ADAM	93.60	2.1 ± 0.05
ADAA	93.37	2.4 ± 0.10
Acarbose	64.23	377.0 ± 1.06

ADA: *A. dhofarica*; H, n-hexane; E, ethyl acetate; Aq, aqueous; M, methanol. Acarbose = positive control; *n*: 3. Data were taken as mean ± SEM; % inhibition at 0.5 mg/mL.

**Table 9:** % Viability and inhibition of metal complexes on breast cancer cell line MDA-MB-231.

Extracts	Conc. (mg/mL)	% Viability	% Inhibition	IC_50_ Values (mg/mL)
ADAM	1.25	98.57	1.42	8.2 ± 0.4
	2.5	92.94	7.05	
	5	67.69	32.30	
	10	39.53	60.46	
ADAA	1.25	103.99	−3.99	NA
	2.5	110.7	−10.77	
	5	107.85	−7.85	
	10	89.91	10.08	
ADAE	1.25	96.17	3.82	6.2 ± 0.3
	2.5	83.54	16.44	
	5	59.24	40.75	
	10	30.87	69.12	
ADAH	1.25	97.53	2.46	8.8 ± 0.6
	2.5	87.72	12.27	
	5	66.13	33.86	
	10	45.27	54.72	
ADAC	1.25	96.90	3.09	7.2 ± 0.4
	2.5	86.99	13.00	
	5	79.69	20.30	
	10	37.55	62.44	

ADA: *A. dhofarica*; H, n-hexane; E, ethyl acetate; A, aqueous; M, methanol. *n*: 3. Data were taken as mean ± SEM.

**Table 10:** Percent viability and percent inhibition capacity of plant extracts on normal breast cell line MCF-10A.

Extracts	Conc. (mg/mL)	% Viability	% Inhibition
ADAM	1.25	96.15	3.84
	2.5	93.66	6.33
	5	82.46	17.53
	10	80.57	19.42
ADAA	1.25	93.18	6.81
	2.5	94.64	5.35
	5	89.50	10.49
	10	80.79	19.20
ADAE	1.25	97.67	2.32
	2.5	94.20	5.79
	5	85.55	14.44
	10	78.46	21.53
ADAH	1.25	94.64	5.35
	2.5	95.29	4.70
	5	89.12	10.87
	10	84.95	15.04
ADAC	1.25	95.50	4.49
	2.5	91.88	8.11
	5	86.79	13.20
	10	79.38	20.61

ADA: *A. dhofarica*, H: n-hexane, E: ethyl acetate, A: aqueous, M: methanol; *n*: 3 with *p* ≤ 0.0I.

**Table 11:** Percent mortality at different concentrations (μg/mL) of *A. dhofarica*.

	Mean % Mortality of Brine Shrimp Larvae (nauplii)			
Concentrations	10	100	250	500
ADAH	0	10 ± 0.09	20 ± 0.3	30 ± 0.3
ADAE	10 ± 0.1	30 ± 0.1	50 ± 0.5	70 ± 0.2
ADAC	0	20 ± 0.1	30 ± 0.2	40 ± 0.5
ADAM	10 ± 0.2	20 ± 0.2	40 ± 0.5	60 ± 0.1
ADAA	10 ± 0.07	20 ± 0.4	30 ± 0.3	50 ± 0.6
KMNO_4_ (Positive control)	30 ± 0.4	40 ± 0.3	60 ± 0.7	80 ± 0.5
DMSO (Negative control)	0	0	0	0

ADA: *A. dhofarica*, M: methanol, H: n-hexane, C: chloroform, A: aqueous KMNO_4_ = positive control. *n*: 3 with *p* ≤ 0.01; data were taken as mean ± SEM.

## Data Availability

The data presented in this study are available in the article.

## Supplementary Materials

The following supporting information can be downloaded at: https://www.mdpi.com/article/10.3390/antibiotics12020354/s1, Table S1. Calculation of Total Phenolic Contents; Table S2. Calculation of Total Flavonoid Contents; Figure S1. Standard calibration curve for Gallic acid; Figure S2. Standard calibration curve for Quercetin; Figure S3. Full-scan (HR-ESI-MS) mass spectrum of the ADAE extract of *A. dhofarica* (negative ionization mode); Figure S4. Full-scan (HR-ESI-MS) mass spectrum of the ADAM extract of *A. dhofarica* (negative ionization mode); Figure S5. HR-ESI-MS mass spectrum ranged 100–525 *m*/*z* of the ADAE extract of *A. dhofarica* (negative ionization mode); Figure S6. Mass spectrum ranged 100–725 *m*/*z* of the ADAM extract of *A. dhofarica* (negative ionization mode).
