# Supplementary material for: Evaluation of Antiproliferative, Antimicrobial, Antioxidant, Antidiabetic and Phytochemical Analysis of Anogeissus dhofarica A. J. Scott
Source: Antibiotics (Basel). 2023 Feb 8;12(2):354. doi: 10.3390/antibiotics12020354 (PMC9952305; doi:10.3390/antibiotics12020354)
Supplement: Supplementary file 1 [file antibiotics-12-00354-s001.zip › antibiotics-2155172-supplementary.pdf]

Supporting Information

Evaluation of Antiproliferative, Antimicrobial, Antioxidant, Antidiabetic and Phytochemical Analysis of *Anogeissus dhofarica* A. J. Scott.

Rabia Maqsood [1,2], Faizullah Khan [1,3], Saeed Ullah [1], Ajmal Khan [1], Habib Al-Jahdhami [1], Javid Hussain [4], Afaf M. Weli [5], Danial Maqsood [6], Shaikh Mizanoor Rahman [1], Amjad Hussain [2,*], Najeeb Ur Rehman [1,*] and Ahmed Al-Harrasi [1,*]

[1] Natural & Medical Sciences Research Center, University of Nizwa, Nizwa 616, Oman; rabia@unizwa.edu.om (R.M); faizullah@unizwa.edu.om (F.K); saidkhan@unizwa.edu.om (S.K); ajmalkhan@unizwa.edu.om (A.K); habib.aljahdhami@unizwa.edu.om (H.A.-J); shaikh.rahman@unizwa.edu.om (S.M.R.)
[2] Department of Chemistry, University of Okara, Okara, Pakistan.
[3] Department of Pharmacy, Abdul Wali Khan University Mardan, Mardan 23200, Pakistan
[4] Department of Biological Sciences & Chemistry, College of Arts and Sciences, University of Nizwa, Oman; javidhej@unizwa.edu.om (J.H.)
[5] School of Pharmacy, College of Pharmacy and Nursing, University of Nizwa, Oman; afaf@unizwa.edu.om (A.M.W.).
[6] Department of Chemistry, University of Lahore, Lahore, Pakistan; 70138167@student.uol.edu.pk (D.M).
[*] Correspondence emails: amjadhussain@uo.edu.pk (A.H); najeeb@unizwa.edu.om (N.U.R), aharrasi@unizwa.edu.om (A. A.-H.).

Table S1. Calculation of Total Phenolic Contents

Extracts	Sample Solution (µg/mL)	Weight of dry extract (g/ml)	Average Absorbance	GAE conc. C (µg/mL)	GAE conc. (mg/ml)	TPC as GAE (mg/ml) $A = \frac{c \times v}{m}$	Mean ± SEM
ADAH	1000	0.001	0.503	83.111	0.083	83.111	0.57
ADAC	1000	0.001	0.903	299.98	0.299	299.98	1.94
ADAE	1000	0.001	0.62	148.23	0.148	148.23	0.57
ADAM	1000	0.001	1.1101	420.45	0.420	420.45	0.57
ADAAq	1000	0.001	0.723	210.86	0.210	210.86	1.20

Table S2. Calculation of Total Flavonoid Contents**.**

**Extracts**	**Sample Solution (µg/mL)**	**Weight of dry extract (g/ml)**	**Average Absorbance**	**QE conc. C (µg/mL)**	**QE conc. (mg/ml)**	**TPC as QE (mg/g)** $A = \frac{c \times v}{m}$	**Mean ± SEM**
ADAH	1000	0.001	0.403	34.4	0.034	34.4	0.4
ADAC	1000	0.001	0.503	80.2	0.080	80.2	0.1
ADAE	1000	0.001	0.542	56.2	0.056	56.2	0.3
ADAM	1000	0.001	0.610	94.1	0.094	94.1	0.3
ADAAq	1000	0.001	0.672	69.6	0.069	69.6	0.2

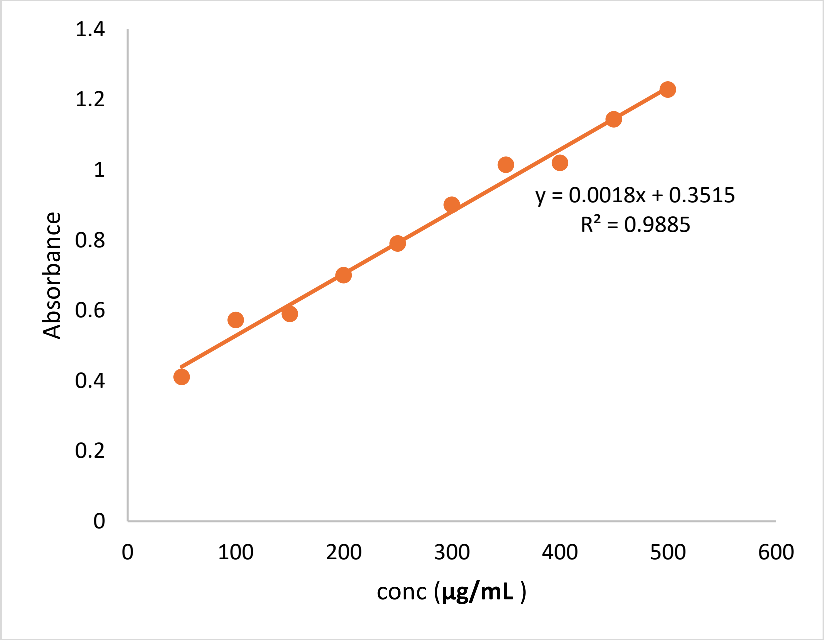

Figure S1. Standard calibration curve for Gallic acid

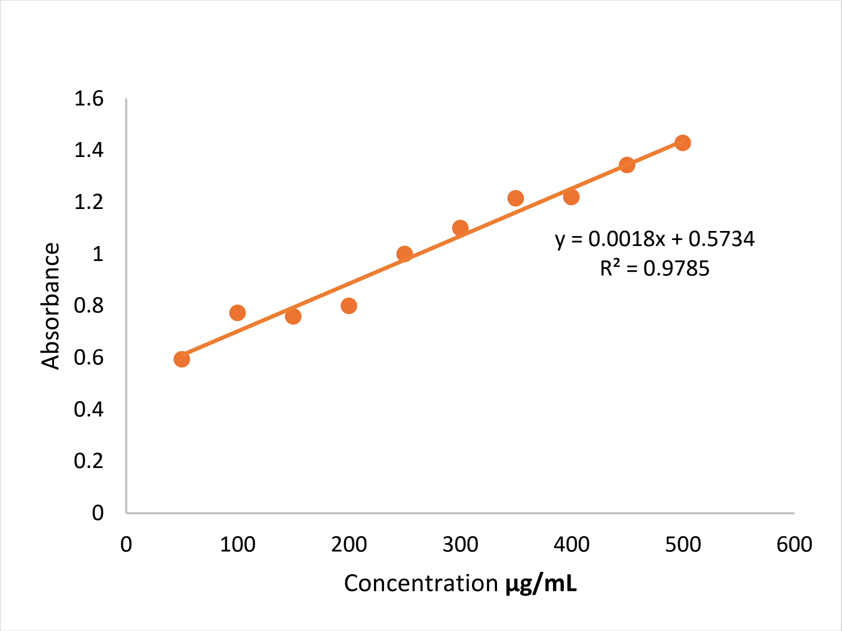

Figure S2. Standard calibration curve for Quercetin

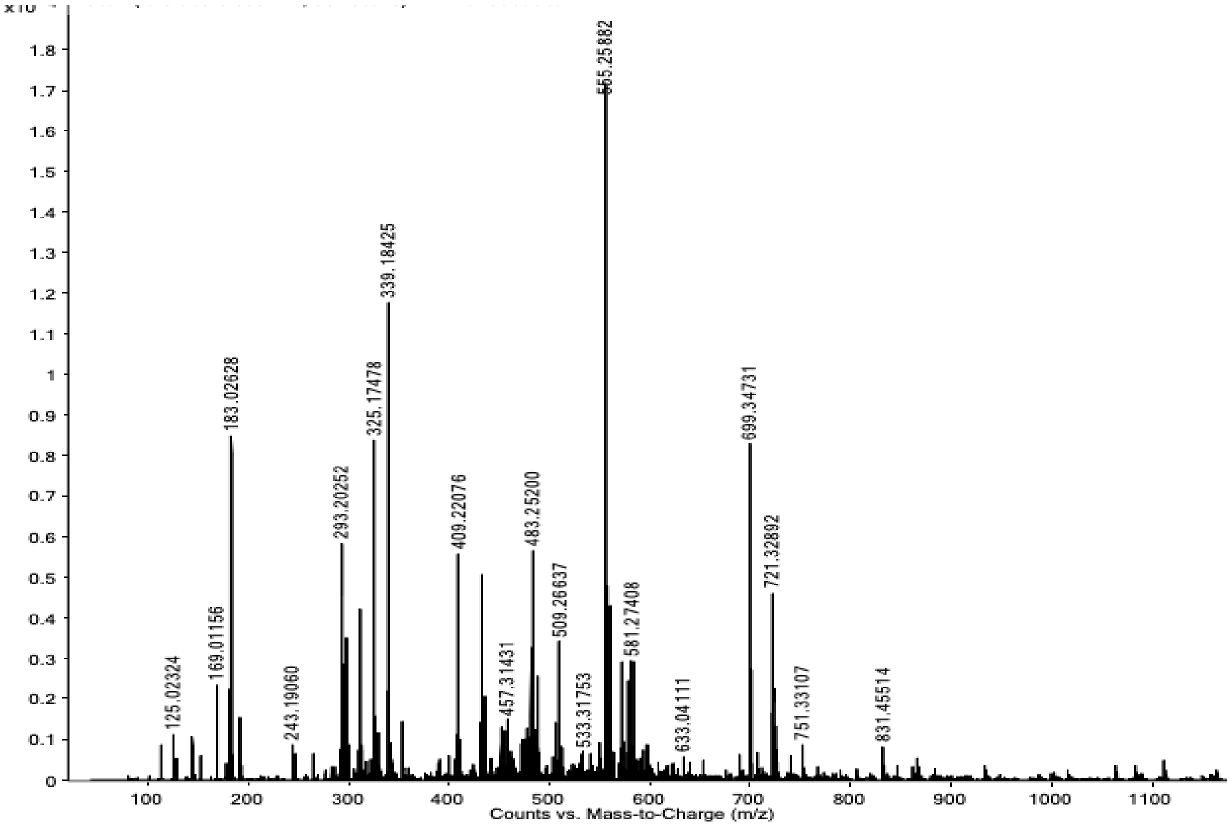

Figure S3. Full-scan (HR-ESI-MS) mass spectrum of the ADAE extract of *A. dhofarica* (negative ionization mode).

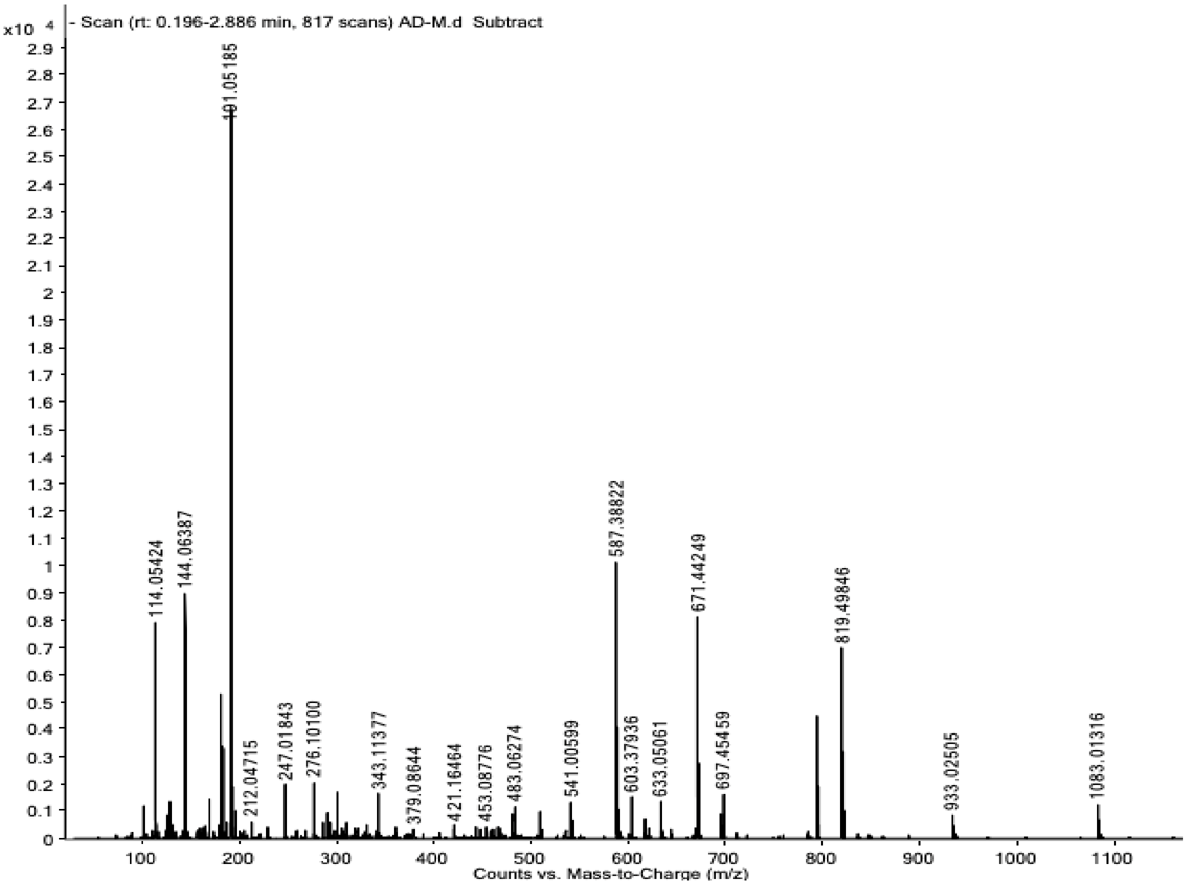

Figure S4. Full-scan (HR-ESI-MS) mass spectrum of the ADAM extract of *A. dhofarica* (negative ionization mode).

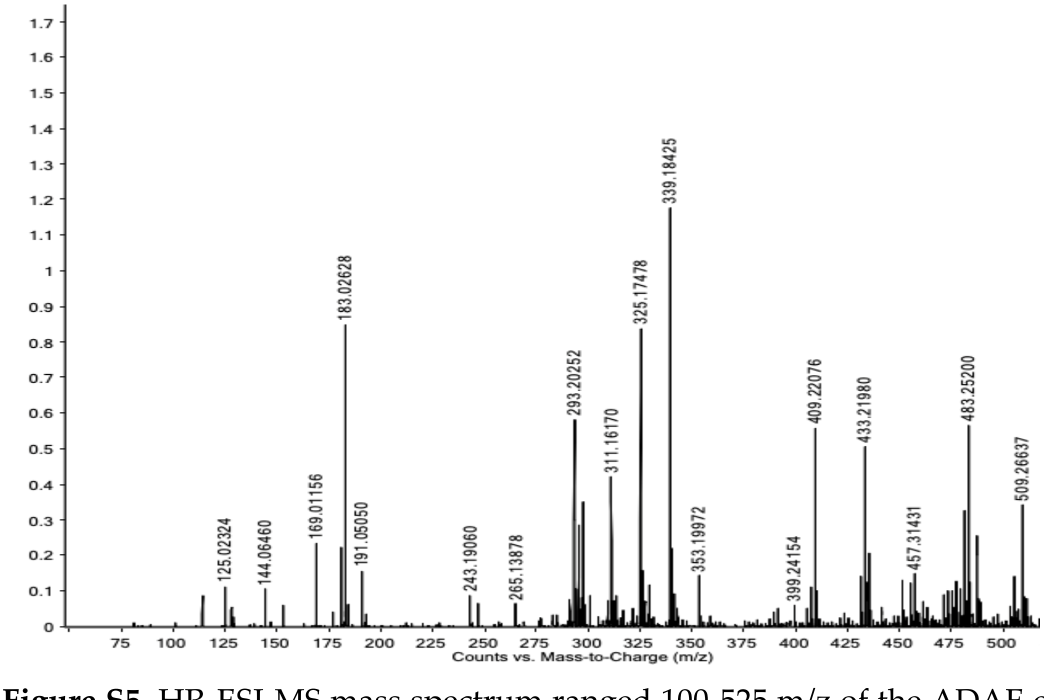

Figure S5. HR-ESI-MS mass spectrum ranged 100-525 m/z of the ADAE extract of *A. dhofarica* (negative ionization mode)

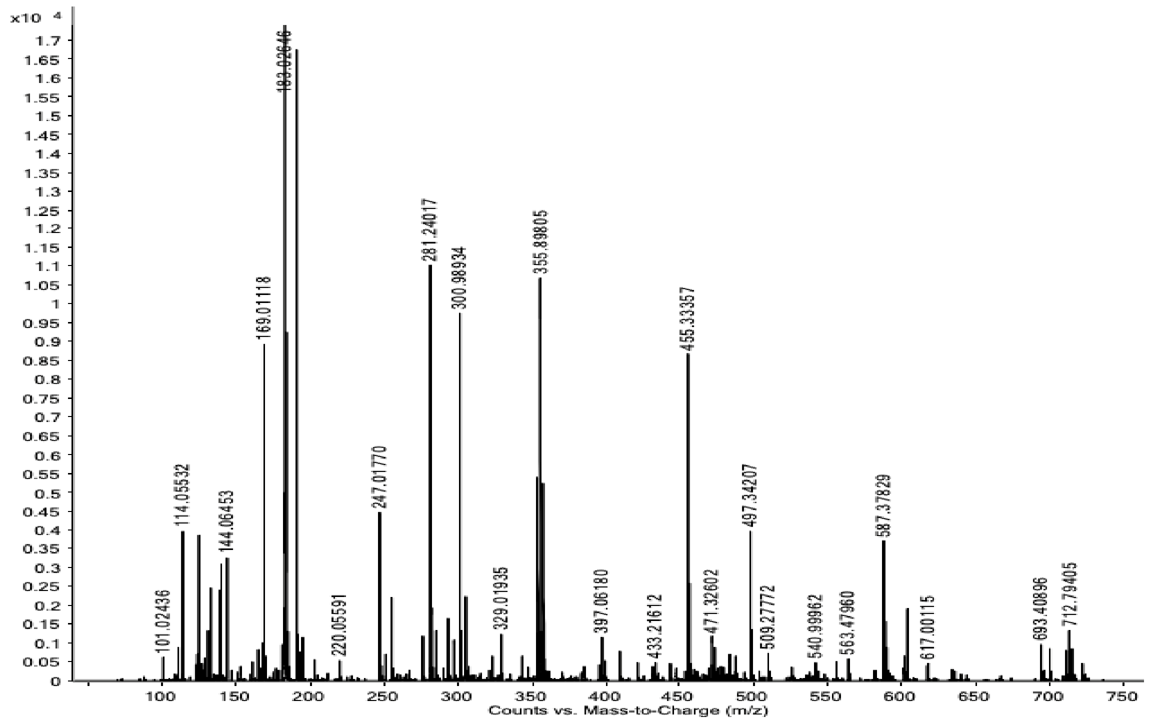

Figure S6. Mass spectrum ranged 100-725 m/z of the ADAM extract of *A. dhofarica* (negative ionization mode).
